# Modeling of free vibrations and resonant frequencies of simply-supported submerged horizontal plate

**DOI:** 10.1371/journal.pone.0298290

**Published:** 2024-03-01

**Authors:** Justyna Slawinska-Budzich, Wojciech Sulisz, Jaroslaw Przewlocki

**Affiliations:** 1 Institute of Hydro-Engineering, Polish Academy of Sciences, Gdansk, Poland; 2 Faculty of Architecture, Gdansk University of Technology, Gdansk, Poland; COMSATS University Islamabad, PAKISTAN

## Abstract

A theoretical approach was applied to study the vibration of simple-supported submerged horizontal plate. The derived analytical solution was used to determine natural frequencies for a horizontal plate vibrating in fluid. The investigations were conducted for a very wide range of material density and elasticity modulus covering all materials used in engineering practice. Analysis shows that plate vibration frequency decreases with increasing plate width and draft, and decreases with decreasing plate thickness. Moreover, the results show that a substantial effect on vibration of submerged plate has mass of water above plate. The results also show that plate vibration frequency decreases with increasing plate material density and decreases with decreasing elasticity modulus. The dominant factors affecting the vibration of the submerged plate are the plate width, the plate thickness, and elasticity modulus. For moderate and low values of elasticity modulus, vibration frequency is becoming lower than frequency of water waves. This is very important because wave frequencies overlap with the natural plate vibration frequencies, which may lead to resonance and failure of a structure. The problem is that the overlap of plate vibration frequencies and wave frequencies occurs for very wide range of wave and plate parameters. Laboratory experiments confirm theoretical results.

## 1. Introduction

The prediction of free vibrations and resonant frequencies of marine structures is one of the main problems of coastal and offshore engineering. The development of techniques for the modelling and prediction of the vibration and resonant frequencies of structures is necessary to ensure the safety and reliability of the entire structures or their elements. The problem is also of vital importance for the maintenance maritime structures. Constructions submerged in seas, such as breakwaters, containers, pipelines, etc. are at risk of being damaged by the attack of waves.

This kind of destruction depends on many factors including type of wave, submergence depth, the geometry of structure, elastic properties of structure elements, etc. This study is devoted to the theoretical investigations of the vibration of an elastic plate submerged in water and prediction of resonant frequencies by taking into account the parameters affecting this phenomenon.

One of the first studies on wave attack on submerged structures were conducted by Brater et al. [1]. They determined wave loads on submerged structures arising from wave attack. Experimental studies were carried out depending on various wave factors. Hayatdavoodi and Ertekin [2] investigated wave-induced pressures acting on a submerged plate. The study comprises wave loads arising from solitary and cnoidal waves. In the following studies Hayatdavoodi and Ertekin [3] and Hayatdavoodi et al. [4] focused on the attack of gravity waves on a thin submerged plate and calculated wave-induced vertical and horizontal forces.

The pressure distribution on a submerged horizontal plate was experimentally and theoretically investigated in [5]. The comparison of the theoretical results with experimental data for the interaction of water waves with a submerged plate is presented in [6, 7]. The vibrations of an elastic plate located on the water surface are investigated in [8].

Numerous theoretical studies have been carried out to analyze the deflection of a submerged horizontal plate. At first, the reflective properties of submerged horizontal plates were studied using the linear potential flow theory. Using the method of matched asymptotic expansions, [9] presented an analytical solution of the reflection and transmission coefficients for long incident waves over an infinitesimal, thin plate submerged in water of constant depth. Based on this work, [10] developed a simplified solution that can be applied to short waves. He showed that the wave reflection at a submerged horizontal plate in shallow water depends on the length of a plate to the wavelength ratio, water depth to the incident wavelength ratio, and the submergence depth of a plate. The derived model provides a reasonable prediction of the transmission and reflection coefficients, however, wave loads were not calculated. Therefore, more complex physical and numerical models are necessary. [11] compared experimental data with numerical solutions obtained for the interaction of regular and random waves with a submerged horizontal thin plate by applying a finite-element method and using the linear-wave diffraction theory.

Wave motion characteristics and flexural modes were studied in finite water depths by applying the generalized dispersion relation. The eigenfunction matching technique and the Wiener-Hopf and Residue Calculus methods were widely used in the study of water-wave interactions with semi-infinite floating elastic plates [12, 13]. In the case of solutions for floating elastic plates of finite length, the problem was solved by applying semi-analytical solutions [14, 15], the Wiener-Hopf and Residue Calculus methods [16], and Green’s functions [17, 18].

While the problem of floating elastic plates has been well studied because of numerous practical applications and interest of oceanographers and geophysicists in the problem of wave scattering by sea ice, much less attention has been given to a situation when the plate is submerged. Pioneering work on this problem was conducted in [19]. In that paper, the problem of a semi-infinite, finite, and circular submerged elastic plate has been investigated by using the eigenfunction-matching method. Another solution for the submerged elastic semi-infinite plate based on the Wiener-Hopf [WH] and Residue Calculus [RC] solutions has been presented in [20]. The impact of fluid flow and vibration on the acoustics of a subsonic flow is examined in [21]. The analysis of wave diffraction in an anisotropic medium is presented in [22, 23]. Recently, experimental investigations of wave-induced deflection of a submerged rectangular cylinder of elastic bottom were conducted in [24]. Laboratory studies of the impact of non-breaking waves on a horizontal emerged deck are in [25].

In the present study, the eigenfunction expansion technique is applied to derive an analytical solution for the vibration of a simply-supported horizontal submerged plate. First, the boundary-value problem is formulated and solved by applying the method of matched eigenfunction expansions. The derived analytical solutions were applied to determine flow fields and loads on a vibrating plate. The derived forces were applied in a motion equation to predict natural frequencies for a simple-supported horizontal plate vibrating in a fluid. Then, laboratory experiments are described and experimental data are presented. Finally, comparisons between theoretical results and experimental data are shown and conclusions are specified.

## 2. Theoretical formulation

### 2.1 Governing equations

**Fig 1:**
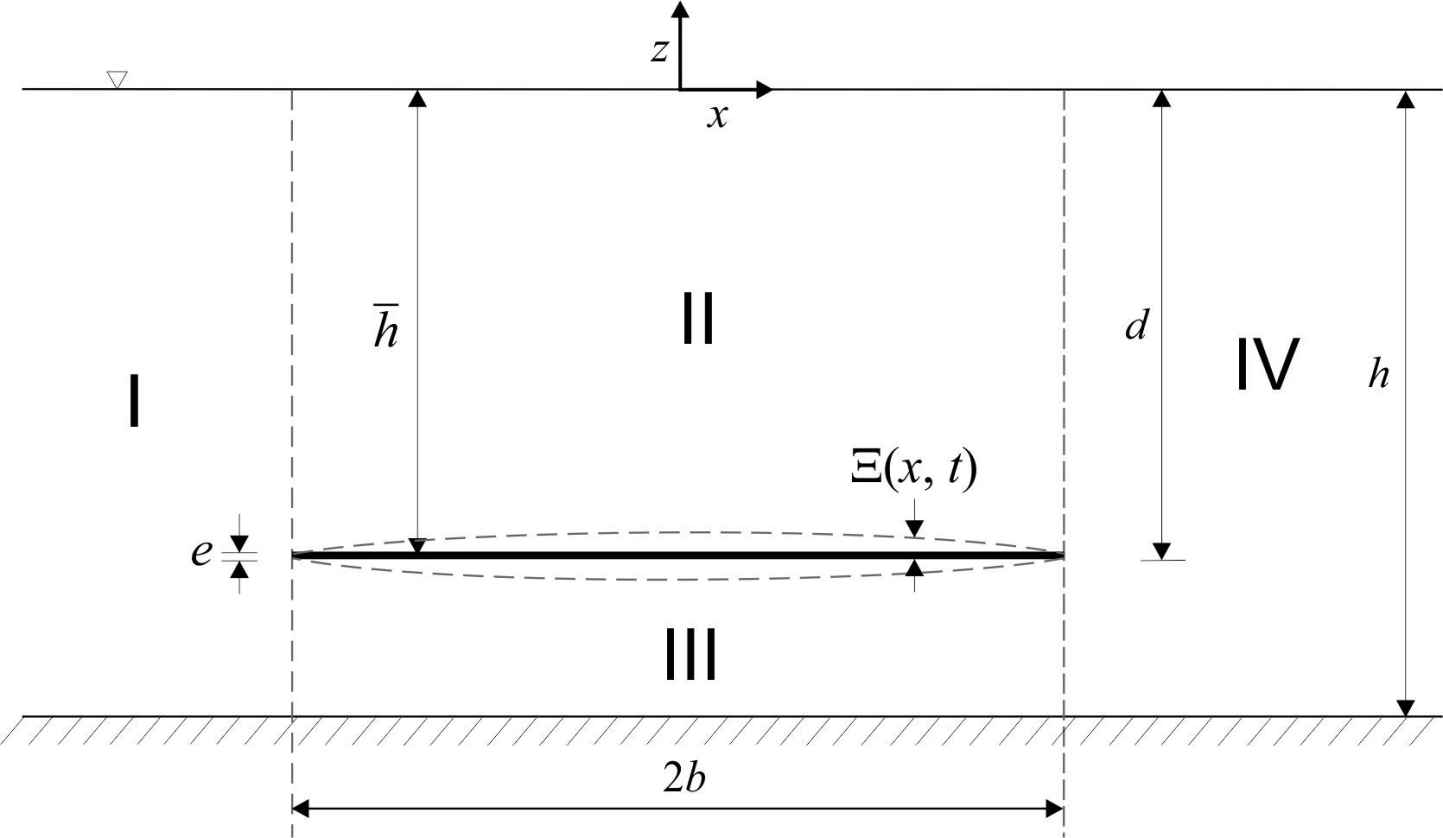
Definitions sketch and coordinate systems.

The problem of the free vibrations of a horizontal plate in the ideal fluid is investigated. The situation under investigation is shown schematically in Fig 1. The problem is defined in the right-hand Cartesian coordinate system selected such that the *xy* plane is horizontal and coincides with the undisturbed free surface and *z* points vertically upwards. It is assumed that

- The fluid is inviscid and incompressible.
- The motion is irrotational.
- The sea bottom and the plate are impervious.

According to the assumptions the fluid motion is governed by a set of equations for the incompressible fluid, namely, the continuity equation

∇⋅V=0 (1a)

and the motion equation

$$V_{t}+\frac{1}{\rho }\nabla (P+\rho gz)=0$$ (1b)

where ***V*** is the fluid velocity vector, ∇ is the two-dimensional vector differential operator, *ρ* is the fluid density, *P* is the pressure, *g* is the acceleration due to gravity.

A submerged plate of width 2*b* is located in water of constant depth *h*. The submergence of the plate is denoted by $\hat{h}$ and the distance from the water surface to the bottom of the plate is *d*. The problem is described in two-dimensional Cartesian coordinates *x*, *z* with the vertical *z-*axis directed vertically upwards from an origin at the still water level. The geometry of the problem is shown schematically in Fig 1.

The boundary-value problem for the velocity potential can be described by the set of the following equations:

$$\nabla^{2}\Phi =0$$ (2a)

at the free surface the velocity potential, Φ (*x*, *z*, *t*), has to satisfy the dynamic boundary condition

Φ=0,z=0 (2b)

at the intersection of the plate and a fluid, a kinematic boundary condition must be fulfilled

$$\Phi_{z}=\Xi_{t}\;,\;|\text{x}|\leq \text{b},\text{z}=-\hat{h},-d$$ (2c)

at the sea bottom, the following boundary condition must be satisfied

$$\Phi_{z}=0,\;z=-h$$ (2d)

moreover, additional boundary conditions are required at infinity

Φ→0,x→±∞ (2e)

where Ξ(x,t) is the deflection of a plate.

The velocity potential, pressure, and deflection of a plate may be expressed as real parts of the following expressions in brackets

$$\Phi (x,z,t)=\text{Re}[\varphi (x,z)e^{-i\omega t}]$$ (3a)

$$P(x,z,t)=\text{Re}[p(x,z)e^{-i\omega t}]$$ (3b)

$$\Xi (x,t)=\text{Re}[\xi (x)e^{-i\omega t}]$$ (3c)

where the quantities in brackets are complex-valued spatial functions and *i =* √-1.

By applying Eq 3a, 3b and 3C and an elastic thin plate theory, the governing equation describing the plate deflection may be written in the following form

$$D\frac{\partial^{4}\xi (x)}{\partial x^{4}}-m\omega^{2}\xi (x)=p(x,-d)-p(x,-\hat{h}),\;|x|\leq b$$ (4)

where *D* = *Ee*^3^/12(1-*ν*^2^) is the flexural plate rigidity, *E* is the modulus of elasticity, *e* is the plate thickness, *ν* is the Poisson ratio, and *m* is the mass per unit width.

### 2.2. Solution technique

The plate deflection may be expanded in a set of modal functions

$$\xi =\sum_{r=1}E_{r}\sin\;\beta_{r}(x+b),\;|x|\leq b\;$$ (5a)

where *E*_*r*_ is the amplitude of the r-th modal function and the eigenvalues must satisfy the following relations

$$\beta_{r}=\frac{r\pi }{2b},r\geq 1$$ (5b)

A velocity potential can be found by dividing the fluid domain into four subdomains R_1_, R_2_, R_3_, and R_4_ where R_1_: *x* ≤ -*b*, -*h* ≤ *z* ≤ 0; R_2_: |*x*| ≤ *b*, -$\hat{h}$ ≤ *z* ≤ 0; R_3_: |*x*| ≤ *b*, -*h* ≤ *z* ≤ -*d*; R_4_: *x* ≥ *b*, -*h* ≤ *z* ≤ 0 and determining a velocity potential in each subdomain. The velocity potentials in each subdomain are determined in the form of eigenfunction expansions.

The velocity potentials in the semi-infinite domains may be written in the following form:

$$\phi_{1}=-\frac{ig}{\omega }\sum_{m=1}R_{rm}\cos\;\alpha_{m}(z+h)e^{\alpha_{m}(x+b)},x\leq -b$$ (6a)

where *R*_*rm*_, *m* = 1,2, …, are the coefficients of the evanescent modes.

The eigenvalues must satisfy the following relations

$$\alpha_{m}=\frac{2m-1}{2h}\pi ,m\geq 1,$$ (6b)

The velocity potential in the sub-domain above the plate may be written in the following form:

$$\phi_{2}=\phi_{21}+\phi_{22},\;|x|\leq b$$ (7a)

where

$$\phi_{21}=-\frac{ig}{\omega }\sum_{m=1}(A_{rm}e^{{\bar{\alpha }}_{m}x}+B_{rm}e^{-{\bar{\alpha }}_{m}x})\cos{\bar{\alpha }}_{m}(z+\hat{h}),\;|x|\leq b$$ (7b)

$$\begin{array}{l} \phi_{22}=-i\omega \sum_{r=1}\frac{E_{r}}{\beta_{r}}\sinh\beta_{r}(z\;+\hat{h})\sin\beta_{r}(x+b)\\ +i\omega \sum_{r=1}\frac{E_{r}\tanh\beta_{r}\hat{h}}{\beta_{r}}\cosh\beta_{r}(z\;+\hat{h})\sin\beta_{r}(x+b),\;|x|\leq b \end{array}$$

in which *A*_*rm*_ and *B*_*rm*_, *m* = 1,2, …, are the coefficients of evanescent modes in the sub-domain above the plate, *E*_*r*_ are the amplitudes of the r-th deflection mode.

The eigenvalues must satisfy the following relations:

$${\bar{\alpha }}_{m}=\frac{2m-1}{2\hat{h}}\pi ,m\geq 1,$$ (7c)

and

$$\beta_{r}=\frac{r\pi }{2b},r\geq 1$$ (7d)

The velocity potential in the sub-domain below the plate may be written in the following form:

$$\phi_{3}=\phi_{31}+\phi_{32},\;|x|\leq b$$ (8a)

where

$$\phi_{31}=-\frac{ig}{\omega }\sum_{n=1}[C_{rm}(1-\delta_{1m}+\delta_{1m}x/b)e^{\mu_{m}x}+D_{rm}e^{-\mu_{m}x}]\cos\mu_{m}(z+h),\;|x|\leq b$$ (8b)

$$\phi_{32}=-i\omega \sum_{r=1}\frac{E_{r}}{\beta_{r}}\frac{\cosh\beta_{r}(z+h)\;}{\sinh\beta_{r}(h-d)}\sin\beta_{r}(x+b),\;|x|\leq b$$

in which *C*_*rm*_ and *D*_*rm*_, *m* = 1, 2, …, are the coefficients of the velocity potential in the sub-domain underneath the plate and the eigenvalues must satisfy the following relations:

$$\mu_{m}=\frac{(m-1)\pi }{h-d},m\geq 1$$ (8c)

The velocity potentials in the semi-infinite domain after the structure may be written in the following form:

$$\phi_{4}=-\frac{ig}{\omega }\sum_{m=1}T_{rm}\cos\;\alpha_{m}(z+h)e^{-\alpha_{m}(x-b)},x\geq b$$ (9)

where *T*_*rm*_, *m* = 1,2, …, are the coefficients of the evanescent modes.

The derived velocity potentials describe standing-type wave fields. The analysis of the velocity potential shows that the velocity and pressure fields decay exponentially from the vibrating plate.

Matching conditions are

$$\phi_{1x}=\left\{\begin{array}{c} \begin{array}{l} \phi_{2x},\;-\hat{h}\leq z\leq 0\\ 0,\;-d\leq z\leq -\hat{h} \end{array}\\ \phi_{3x},-h\leq z\leq -d \end{array}\right.,\;x=-b$$ (10a)

$$\begin{array}{l} \phi_{1}=\phi_{2},-\hat{h}\leq z\leq 0\\ \phi_{1}=\phi_{3},-h\leq z\leq -d \end{array}$$ (10b)

$$\phi_{4x}=\left\{\begin{array}{c} \begin{array}{l} \phi_{2x},\;-\hat{h}\leq z\leq 0\\ 0,\;-d\leq z\leq -\hat{h} \end{array}\\ \phi_{3x},-h\leq z\leq -d \end{array}\right.,\;x=b$$ (10c)

$$\begin{array}{l} \phi_{4}=\phi_{2},-\hat{h}\leq z\leq 0\\ \phi_{4}=\phi_{3},-h\leq z\leq -d \end{array}$$ (10d)

The coefficients of the velocity potential in the subdomain before the plate may be derived from Eq 10a that may be written in the following form:

$$\int_{-h}^{-d}\phi_{1x}\cos\;\alpha_{m}(z+h)dz=\int_{-\hat{h}}^{0}\left(\phi_{21x}+\phi_{22x}\right)\cos\;\alpha_{m}(z+h)dz+\int_{-h}^{-d}\left(\phi_{31x}+\phi_{32x}\right)\cos\;\alpha_{m}(z+h)dz,$$

Taking into account Eqs 6a, 7a, and 8a one obtains

$$\begin{array}{l} R_{rm}=\frac{1}{\alpha_{m}\int_{-h}^{0}\cos^{2}\alpha_{m}\left(z+h\right)dz}\\ \text{\{}\varepsilon_{2}\;\sum_{l=1}(A_{rl}{\bar{\alpha }}_{l}e^{-{\bar{\alpha }}_{l}b}-B_{rl}{\bar{\alpha }}_{l}e^{{\bar{\alpha }}_{l}b})\int_{-\hat{h}}^{0}\cos{\bar{\alpha }}_{l}\left(z+\hat{h}\right)\cos\;\alpha_{m}\left(z+h\right)dz\\ +\varepsilon_{3}\;\sum_{l=1}\left[C_{rl}\left(\mu_{l}+\delta_{1l}/b\right)e^{-\mu_{l}b}-D_{rl}\mu_{l}e^{\mu_{l}b}\right]\int_{-h}^{-d}\cos\mu_{l}(z+h)\cos\;\alpha_{m}(z+h)dz\\ +\frac{i\omega }{g}\left(\int_{-\hat{h}}^{0}\phi_{22x}\cos\;\alpha_{m}(z+h)dz+\int_{-h}^{-d}\phi_{32x}\cos\;\alpha_{m}(z+h)dz\right)\},m=1,\;2,.. \end{array}$$ (11a)

which results in

$$\begin{array}{l} R_{rm}=\frac{4}{2\alpha_{m}h+\sin2\alpha_{m}h}\\ \text{\{}\varepsilon_{2}\;\sum_{l=1}(A_{rl}{\bar{\alpha }}_{l}e^{-{\bar{\alpha }}_{l}b}-B_{rl}{\bar{\alpha }}_{l}e^{{\bar{\alpha }}_{l}b}){\left[\frac{\sin\left({\bar{\alpha }}_{l}\left(z+\hat{h}\right)-\alpha_{m}\left(z+h\right)\right)}{2\left({\bar{\alpha }}_{l}-\alpha_{m}\right)}+\frac{\sin\left({\bar{\alpha }}_{l}\left(z+\hat{h}\right)+\alpha_{m}\left(z+h\right)\right)}{2\left({\bar{\alpha }}_{l}+\alpha_{m}\right)}\right]}_{-\hat{h}}^{0}\\ +\varepsilon_{3}\;\sum_{l=1}\left[C_{rl}\left(\mu_{l}+\delta_{1l}/b\right)e^{-\mu_{l}b}-D_{rl}\mu_{l}e^{\mu_{l}b}\right]\;{\left[\frac{\sin\left(\mu_{l}\left(z+h\right)-\alpha_{m}\left(z+h\right)\right)}{2\left(\mu_{l}-\alpha_{m}\right)}+\frac{\sin\left(\mu_{l}\left(z+h\right)+\alpha_{m}\left(z+h\right)\right)}{2\left(\mu_{l}-\alpha_{m}\right)}\right]}_{-h}^{-d}\\ +\frac{i\omega }{g}\left(\int_{-\hat{h}}^{0}\phi_{22x}\cos\;\alpha_{m}(z+h)dz+\int_{-h}^{-d}\phi_{32x}\cos\;\alpha_{m}(z+h)dz\right)\text{\}},\\ m=1,\;2,.. \end{array}$$ (11b)

or

$$\begin{array}{l} R_{rm}=\frac{4}{2\alpha_{m}h+\text{sin}2\alpha_{m}h}\\ \text{\{}\varepsilon_{2}\;\sum_{l=1}(A_{rl}{\bar{\alpha }}_{l}e^{-{\bar{\alpha }}_{l}b}-B_{rl}{\bar{\alpha }}_{l}e^{{\bar{\alpha }}_{l}b}\text{)[}\frac{\text{sin}({\bar{\alpha }}_{l}\hat{h}-\alpha_{m}h\text{)}+\text{sin}\alpha_{m}(h-\hat{h}\text{)}}{2({\bar{\alpha }}_{l}-\alpha_{m})}+\frac{\text{sin}({\bar{\alpha }}_{l}\hat{h}+\alpha_{m}\hat{h}\text{)}-\text{sin}\alpha_{m}(h-\hat{h}\text{)}}{2({\bar{\alpha }}_{l}+\alpha_{m}\text{)}}\text{]}\\ +\varepsilon_{3}\;\sum_{l=1}\text{[}C_{rl}(\mu_{l}+\delta_{1l}/b\text{)}e^{-\mu_{l}b}-D_{rl}\mu_{l}e^{\mu_{l}b}\text{][}\frac{\text{sin}(h-d\text{)(}\alpha_{m}-\mu_{l}\text{)}}{2(\mu_{l}-\alpha_{m}\text{)}}+\frac{\text{sin}(h-d\text{)(}\alpha_{m}+\mu_{l}\text{)}}{2(\mu_{l}+\alpha_{m}\text{)}}\text{]}\\ +\frac{i\omega }{g}\left(\int_{-\hat{h}}^{0}\phi_{22x}\cos\;\alpha_{m}(z+h)dz+\int_{-h}^{-d}\phi_{32x}\cos\;\alpha_{m}(z+h)dz\right)\text{\},}\\ m=1,\;2,.. \end{array}$$ (11c)

The integrations

$$\int_{-\hat{h}}^{0}\phi_{22x}\cos\;\alpha_{m}(z+h)dz$$ (11d)

$$\;\int_{-h}^{-d}\phi_{32x}\cos\;\alpha_{m}(z+h)dz$$

are conducted analytically and the results are included in S1 Appendix.

The coefficients of the velocity potential in the subdomain after the plate may be derived from Eq 10c that may be written in the following form:

$$\begin{array}{l} \int_{-h}^{-d}\phi_{4x}\cos\;\alpha_{m}(z+h)dz=\int_{-\hat{h}}^{0}\left(\phi_{21x}+\phi_{22x}\right)\cos\;\alpha_{m}(z+h)dz\\ +\int_{-h}^{-d}\left(\phi_{31x}+\phi_{32x}\right)\cos\;\alpha_{m}(z+h)dz \end{array}$$ (12a)

Taking into account Eqs 7b, 8b and 9 one obtains:

$$\begin{array}{l} T_{rm}=-\frac{1}{\alpha_{m}\int_{-h}^{0}\cos^{2}\alpha_{m}(z+h)dz}\\ \text{\{}\varepsilon_{2}\;\sum_{l=1}\left(A_{rl}{\bar{\alpha }}_{l}e^{{\bar{\alpha }}_{l}b}-B_{rl}{\bar{\alpha }}_{l}e^{-{\bar{\alpha }}_{l}b}\right)\int_{-\hat{h}}^{0}\cos{\bar{\alpha }}_{l}\left(z+\hat{h}\right)\cos\;\alpha_{m}\left(z+h\right)dz\\ +\varepsilon_{3}\;\sum_{l=1}\left[C_{rl}\left(\mu_{l}+\delta_{1l}/b\right)e^{\mu_{l}b}-D_{rl}\mu_{l}e^{-\mu_{l}b}\right]\int_{-h}^{-d}\cos\mu_{l}\left(z+h\right)\cos\;\alpha_{m}\left(z+h\right)dz\\ +\frac{i\omega }{g}\left(\int_{-\hat{h}}^{0}\phi_{22x}\cos\;\alpha_{m}(z+h)dz+\int_{-h}^{-d}\phi_{32x}\cos\;\alpha_{m}(z+h)\right)dz\text{\}},m=1,\;2,.. \end{array}$$ (12b)

which results in

$$\begin{array}{l} T_{rm}=-\frac{4}{2\alpha_{m}h+\sin2\alpha_{m}h}\\ \text{\{}\varepsilon_{2}\;\sum_{l=1}\left(A_{rl}{\bar{\alpha }}_{l}e^{{\bar{\alpha }}_{l}b}-B_{rl}{\bar{\alpha }}_{l}e^{-{\bar{\alpha }}_{l}b}\right){\left[\frac{\sin\left({\bar{\alpha }}_{l}\left(z+\hat{h}\right)-\alpha_{m}\left(z+h\right)\right)}{2({\bar{\alpha }}_{l}-\alpha_{1})}+\frac{\sin\left({\bar{\alpha }}_{l}\left(z+\hat{h}\right)+\alpha_{m}\left(z+h\right)\right)}{2({\bar{\alpha }}_{l}+\alpha_{m})}\right]}_{-\hat{h}}^{0}\\ +\varepsilon_{3}\;\sum_{l=1}\left[C_{rl}\left(\mu_{l}+\delta_{1l}/b\right)e^{\mu_{l}b}-D_{rl}\mu_{l}e^{-\mu_{l}b}\right]{\left[\frac{\sin\left(\mu_{l}-\alpha_{m}\right)\left(z+h\right)}{2\left(\mu_{l}-\alpha_{m}\right)}+\frac{\sin\left(\mu_{l}+\alpha_{m}\right)\left(z+h\right)}{2\left(\mu_{l}+\alpha_{m}\right)}\right]}_{-h}^{-d}\\ +\frac{i\omega }{g}\left(\int_{-\hat{h}}^{0}\phi_{22x}\cos\;\alpha_{m}(z+h)dz+\int_{-h}^{-d}\phi_{32x}\cos\;\alpha_{m}(z+h)dz\right)\text{\}},m=1,\;2,.. \end{array}$$ (12c)

or

$$\begin{array}{l} T_{rm}=-\frac{4}{2\alpha_{m}h+\text{sin}2\alpha_{m}h}\\ \text{\{}\varepsilon_{2}\;\sum_{l=1}(A_{rl}{\bar{\alpha }}_{l}e^{{\bar{\alpha }}_{l}b}-B_{rl}{\bar{\alpha }}_{l}e^{-{\bar{\alpha }}_{l}b}\text{)[}\frac{\text{sin}({\bar{\alpha }}_{l}\hat{h}-\alpha_{m}h\text{)}+\text{sin}\alpha_{m}(h-\hat{h}\text{)}}{2({\bar{\alpha }}_{l}-\alpha_{m})}+\frac{\text{sin}({\bar{\alpha }}_{l}\hat{h}+\alpha_{m}\hat{h}\text{)}-\text{sin}\alpha_{m}(h-\hat{h}\text{)}}{2({\bar{\alpha }}_{l}+\alpha_{m}\text{)}}\text{]}\\ +\varepsilon_{3}\;\sum_{l=1}\text{[}C_{rl}(\mu_{l}+\delta_{1l}/b\text{)}e^{\mu_{l}b}-D_{rl}\mu_{l}e^{-\mu_{l}b}\text{][}\frac{\text{sin}(h-d\text{)(}\alpha_{m}-\mu_{l}\text{)}}{2(\mu_{l}-\alpha_{m}\text{)}}+\frac{\text{sin}(h-d\text{)(}\alpha_{m}+\mu_{l}\text{)}}{2(\mu_{l}+\alpha_{m}\text{)}}\text{]}\\ +\frac{i\omega }{g}\left(\int_{-\hat{h}}^{0}\phi_{22x}\cos\;\alpha_{m}(z+h)dz+\int_{-h}^{-d}\phi_{32x}\cos\;\alpha_{m}(z+h)dz\right)\text{\},}\\ m=1,\;2,.. \end{array}$$ (12d)

The integrations

$$\int_{-\hat{h}}^{0}\phi_{22x}\cos\;\alpha_{m}(z+h)dz$$ (12e)

$$\;\int_{-h}^{-d}\phi_{32x}\cos\;\alpha_{m}(z+h)dz$$

are conducted analytically and the results are included in S1 Appendix.

The coefficients of the velocity potential in the subdomain above the plate may be derived from Eq 10b and 10d. The first Eq 10b, may be written in the following form:

$$\int_{-\hat{h}}^{0}\phi_{1}\cos{\bar{\alpha }}_{m}\left(z+\hat{h}\right)dz=\int_{-\hat{h}}^{0}\phi_{2}\cos{\bar{\alpha }}_{m}\left(z+\hat{h}\right)dz$$ (13a)

Taking into account Eqs 6 and 7 one obtains

$$\begin{array}{l} \int_{-\hat{h}}^{0}\sum_{l=1}R_{rl}\cos\;\alpha_{l}\left(z+h\right)\cos{\bar{\alpha }}_{m}\left(z+\hat{h}\right)dz=\\ \left(S_{2}+if_{2}\right)\left(A_{rm}e^{-{\bar{\alpha }}_{m}b}+B_{rm}e^{{\bar{\alpha }}_{m}b}\right)\frac{\;2{\bar{\alpha }}_{m}\hat{h}+\sin2{\bar{\alpha }}_{m}\hat{h}}{4{\bar{\alpha }}_{m}}\\ +\frac{i\omega }{g}\int_{-\hat{h}}^{0}\;\phi_{22}\cos{\bar{\alpha }}_{m}\left(z+\hat{h}\right)dz,\;m=1,2,... \end{array}$$ (13b)

which results in

$$\begin{array}{l} \sum_{l=1}R_{rl}[\frac{\text{sin}({\bar{\alpha }}_{m}\hat{h}-\alpha_{l}h\text{)}+\text{sin}\alpha_{l}(h-\hat{h}\text{)}}{2({\bar{\alpha }}_{m}-\alpha_{l})}+\frac{\text{sin}({\bar{\alpha }}_{m}\hat{h}+\alpha_{l}\hat{h})-\text{sin}\alpha_{l}(h-\hat{h}\text{)}}{2({\bar{\alpha }}_{m}+\alpha_{l}\text{)}}]=\\ \left(S_{2}+if_{2}\right)\left(A_{rm}e^{-{\bar{\alpha }}_{m}b}+B_{rm}e^{{\bar{\alpha }}_{m}b}\right)\frac{\;2{\bar{\alpha }}_{m}\hat{h}+\sin2{\bar{\alpha }}_{m}\hat{h}}{4{\bar{\alpha }}_{m}}\\ +\frac{i\omega }{g}\int_{-\hat{h}}^{0}\;\phi_{22}\cos{\bar{\alpha }}_{m}\left(z+\hat{h}\right)dz,\;m=1,2,... \end{array}$$ (13c)

The second Eq 10d, may be written in the following form

$$\int_{-\hat{h}}^{0}\phi_{4}\cos{\bar{\alpha }}_{m}\left(z+\hat{h}\right)dz=\int_{-\hat{h}}^{0}\phi_{2}\cos{\bar{\alpha }}_{m}\left(z+\hat{h}\right)dz$$ (14a)

Taking into account Eqs 7 and 9 one obtains

$$\begin{array}{l} \int_{-\hat{h}}^{0}\sum_{l=1}T_{rl}\cos\;\alpha_{l}\left(z+h\right)\cos{\bar{\alpha }}_{m}\left(z+\hat{h}\right)dz=\\ \left(S_{2}+if_{2}\right)\left(A_{rm}e^{{\bar{\alpha }}_{m}b}+B_{rm}e^{-{\bar{\alpha }}_{m}b}\right)\frac{\;2{\bar{\alpha }}_{m}\hat{h}+\sin2{\bar{\alpha }}_{m}\hat{h}}{4{\bar{\alpha }}_{m}}\\ +\frac{i\omega }{g}\int_{-\hat{h}}^{0}\;\phi_{22}\cos{\bar{\alpha }}_{m}\left(z+\hat{h}\right)dz,,\;m=1,2,... \end{array}$$ (14b)

which results in

$$\begin{array}{l} \sum_{l=1}T_{rl}[\frac{\text{sin}({\bar{\alpha }}_{m}\hat{h}-\alpha_{l}h\text{)}+\text{sin}\alpha_{l}(h-\hat{h}\text{)}}{2({\bar{\alpha }}_{m}-\alpha_{l})}+\frac{\text{sin}({\bar{\alpha }}_{m}\hat{h}+\alpha_{l}\hat{h})-\text{sin}\alpha_{l}(h-\hat{h}\text{)}}{2({\bar{\alpha }}_{m}+\alpha_{l}\text{)}}]=\\ \left(S_{2}+if_{2}\right)\left(A_{rm}e^{{\bar{\alpha }}_{m}b}+B_{rm}e^{-{\bar{\alpha }}_{m}b}\right)\frac{\;2{\bar{\alpha }}_{m}\hat{h}+\sin2{\bar{\alpha }}_{m}\hat{h}}{4{\bar{\alpha }}_{m}}\\ +\frac{i\omega }{g}\int_{-\hat{h}}^{0}\;\phi_{22}\cos{\bar{\alpha }}_{m}\left(z+\hat{h}\right)dz,,\;m=1,2,... \end{array}$$ (14c)

The integrations

$$\int_{-\hat{h}}^{0}\;\phi_{22}\cos{\bar{\alpha }}_{m}\left(z+\hat{h}\right)dz$$ (14d)

are conducted analytically and the results are included in S1 Appendix.

The coefficients of the velocity potential in the subdomain under the plate may be derived from Eq 10b and 10d. The first Eq 10b, may be written in the following form:

$$\int_{-h}^{-d}\phi_{1}\cos\mu_{m}\left(z+h\right)dz=\int_{-h}^{-d}\phi_{3}\cos\mu_{m}\left(z+h\right)dz$$ (15a)

Taking into account Eqs 6 and 8 one obtains

$$\begin{array}{l} \int_{-h}^{-d}\sum_{m=1}R_{rl}\cos\;\alpha_{l}\left(z+h\right)\cos\mu_{m}\left(z+h\right)dz=\\ \left(S_{3}+if_{3}\right)\left[C_{rm}\left(1-\delta_{1m}-\delta_{1m}\right)e^{-\mu_{m}b}+D_{1m}e^{\mu_{m}b}\right]\int_{-h}^{-d}\cos^{2}\mu_{m}\left(z+h\right)dz\\ +\frac{i\omega }{g}\int_{-h}^{-d}\;\phi_{32}\cos\mu_{m}\left(z+h\right)dz,\;m=1,2,... \end{array}$$ (15b)

which results in

$$\begin{array}{l} \sum_{l=1}R_{rl}\text{[}\frac{\text{sin}(h-d\text{)(}\mu_{m}-\alpha_{l}\text{)}}{2(\alpha_{l}-\mu_{m}\text{)}}+\frac{\text{sin}(h-d\text{)(}\mu_{m}+\alpha_{l}\text{)}}{2(\alpha_{l}+\mu_{m}\text{)}}]=\\ \left(S_{3}+if_{3}\right)\left[C_{rm}(1-\delta_{1m}-\delta_{1m})e^{-\mu_{m}b}+D_{rm}e^{\mu_{m}b}\right]\frac{\mu_{m}\left(h-d\right)+\sin2\mu_{m}\left(h-d\right)}{4\mu_{m}}\\ +\frac{i\omega }{g}\int_{-h}^{-d}\;\phi_{32}\cos\mu_{m}\left(z+h\right)dz,\;m=1,2,... \end{array}$$ (15c)

The second Eq 10d, may be written in the following form

$$\int_{-h}^{-d}\phi_{4}\cos\mu_{m}\left(z+h\right)dz=\int_{-h}^{-d}\phi_{3}\cos\mu_{m}\left(z+h\right)dz$$ (16a)

Taking into account Eqs 8 and 9 one obtains

$$\begin{array}{l} \int_{-h}^{-d}\sum_{m=1}T_{rl}\cos\;\alpha_{l}\left(z+h\right)\cos\mu_{m}\left(z+h\right)dz=\\ \left(S_{3}+if_{3}\right)\left[C_{rm}\left(1-\delta_{1m}+\delta_{1m}\right)e^{\mu_{m}b}+D_{rm}e^{-\mu_{m}b})\right]\int_{-h}^{-d}\cos^{2}\mu_{m}\left(z+h\right)dz+\\ +\frac{i\omega }{g}\int_{-h}^{-d}\;\phi_{32}\cos\mu_{m}\left(z+h\right)dz,\;m=1,2,... \end{array}$$ (16b)

By conducting integration, Eq 16b, may be further simplified to the following form

$$\begin{array}{l} \sum_{l=1}T_{rl}\text{[}\frac{\text{sin}(h-d\text{)(}\mu_{m}-\alpha_{l}\text{)}}{2(\alpha_{l}-\mu_{m}\text{)}}+\frac{\text{sin}(h-d\text{)(}\mu_{m}+\alpha_{l}\text{)}}{2(\alpha_{l}+\mu_{m}\text{)}}]=\\ \left(S_{3}+if_{3}\right)\left[C_{rm}\left(1-\delta_{1m}+\delta_{1m}\right)e^{\mu_{m}b}+D_{rm}e^{-\mu_{m}b}\right]\frac{\mu_{m}\left(h-d\right)+\sin2\mu_{m}\left(h-d\right)}{4\mu_{m}}+\\ +\frac{i\omega }{g}\int_{-h}^{-d}\;\phi_{32}\cos\mu_{m}\left(z+h\right)dz,\;m=1,2,... \end{array}$$ (16c)

The integrations

$$\int_{-h}^{-d}\;\phi_{32}\cos\mu_{m}\left(z+h\right)dz$$ (16d)

are conducted analytically and the results are included in S1 Appendix.

The formulation is general and comprises damping in the fluid sub-domains above and below the plate. However, due to a large number of parameters, the analysis of results is conducted for ideal fluid s_2_ = 1, s_3_ = 1, f_2_ = 0, f_3_ = 0, ε_2_ = 1, ε_3_ = 1.

### 2.3. Radiation problem and free vibrations

The derived semi-analytical solution/model is applied to determine velocity potentials for consecutive plate modes of unite amplitudes *E*_*r*_ = 1, *r* = 1, 2,… This enables us to write the equation describing the plate deflection in the following form

$$-\omega^{2}m\;\sum_{r=1}E_{r}\sin\beta_{r}(x+b)+D\sum_{r=1}E_{r}\beta_{r}^{4}\sin\beta_{r}(x+b)=p(x,-d)-p(x,-\hat{h}),|x|\leq b$$ (17a)

where

$$\begin{array}{l} p(x,-\hat{h})=-\rho \omega^{2}\sum_{r=1}\frac{E_{r}\tanh\beta_{r}\hat{h}}{\beta_{r}}\sin\beta_{r}(x+b)+\\ \rho g\sum_{r=1}\sum_{l=1}\frac{E_{l}}{\int_{-b}^{b}\sin^{2}\beta_{r}(x+b)dx}\int_{-b}^{b}\sum_{m=1}(A_{lm}e^{{\bar{\alpha }}_{m}x}+B_{lm}e^{-{\bar{\alpha }}_{m}x})\sin\beta_{r}(x+b)dx\sin\beta_{r}(x+b) \end{array}$$ (17b)

$$\begin{array}{l} p(x,-d)=\rho \omega^{2}\sum_{r=1}\frac{E_{r}}{\beta_{r}}\frac{\cosh\beta_{r}(h-d)\;}{\sinh\beta_{r}(h-d)}\sin\beta_{r}(x+b)+\\ \rho g\sum_{r=1}\sum_{l=1}\frac{E_{l}}{\int_{-b}^{b}\sin^{2}\beta_{r}(x+b)dx}\int_{-b}^{b}\sum_{m=1}[C_{lm}(1-\delta_{1m}+\delta_{1m}x/b)e^{\mu_{m}x}+D_{lm}e^{-\mu_{m}x}](-1{)}^{m-1}\\ \sin\beta_{r}(x+b)dx\;\sin\beta_{r}(x+b) \end{array}$$

The comparisons of the coefficients of the Fourier series lead to the following set of equations

$$\begin{array}{l} -\omega^{2}m\;E_{r}-\rho \omega^{2}\frac{E_{r}}{\beta_{r}}\frac{\cosh\beta_{r}(h-d)\;}{\sinh\beta_{r}(h-d)}-\rho \omega^{2}\frac{E_{r}\tanh\beta_{r}\hat{h}}{\beta_{r}}+DE_{r}\beta_{r}^{4}-\\ \rho g\sum_{l=1}\frac{E_{l}}{\int_{-b}^{b}\sin^{2}\beta_{r}(x+b)dx}\int_{-b}^{b}\sum_{m=1}[C_{lm}(1-\delta_{1m}+\delta_{1m}x/b)e^{\mu_{m}x}+D_{lm}e^{-\mu_{m}x}](-1{)}^{m-1}\sin\beta_{r}(x+b)dx\\ +\rho g\sum_{l=1}\frac{E_{l}}{\int_{-b}^{b}\sin^{2}\beta_{r}(x+b)dx}\int_{-b}^{b}\sum_{m=1}(A_{lm}e^{{\bar{\alpha }}_{m}x}+B_{lm}e^{-{\bar{\alpha }}_{m}x})\sin\beta_{r}(x+b)dx=0,r=1,2.. \end{array}$$ (18)

Eq 18 enables us to determine the free vibration frequencies of a submerged plate. Since the first mode drastically dominates in the free vibrations of a submerged plate, the plate vibration frequency may be determined analytically

$$\begin{array}{l} \omega^{2}=\\ \frac{D\beta_{1}^{4}b+\rho g\int_{-b}^{b}\sum_{m=1}\{A_{1m}e^{{\bar{\alpha }}_{m}x}+B_{1m}e^{-{\bar{\alpha }}_{m}x}-[C_{1m}(1-\delta_{1m}+\delta_{1m}x/b)e^{\mu_{m}x}+D_{1m}e^{-\mu_{m}x}]{\left(-1\right)}^{m-1}\}\sin\beta_{1}(x+b)dx}{b[m+\rho \frac{\cosh\beta_{1}(h-d)\;}{\beta_{1}\sinh\beta_{1}(h-d)}+\rho \frac{\tanh\beta_{1}\hat{h}}{\beta_{1}}]} \end{array}$$ (19)

## 3. Theoretical results

The derived analytical solution was applied to investigate the effect of the geometry and material properties of a simply-supported thin plate on the free vibrations of the plate submerged in water. The calculations are conducted for a wide range of plate frequencies and several important parameters of the model including the length and thickness of a plate, the plate submerged rate as well as the material properties of a plate comprising the material density and stiffness rate.

**Fig 2:**
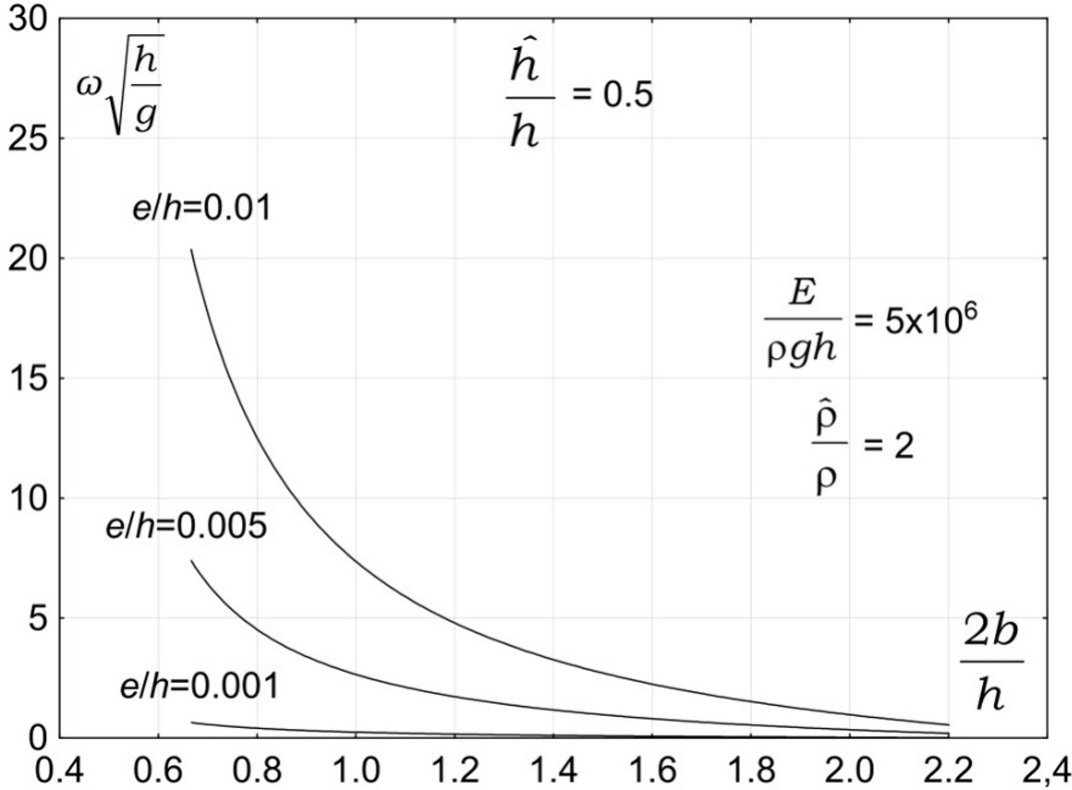
Effect of plate width and thickness on vibration frequency, *E*/*ρ*gh = 5×10^6^$\hat{\rho }/\rho =2$.

The first group of parameters affecting the vibration of a submerged plate is associated with the plate geometry. The geometry of a plate is expected to have a significant effect on the vibration of a submerged plate. A typical dependency of the vibration frequency on the plate geometrical parameters is presented in Fig 2. The plots show the dimensionless vibration frequencies, $\omega \sqrt{h/g}$, plotted versus the dimensionless plate length, 2b/h, for three ratios of the plate thickness to the water depth e/h = 0.01, e/h = 0.005, and e/h = 0.001. The results are plotted for $\hat{h}/h=0.5$, $\hat{\rho }/\rho =2$, and *E*/*ρgh* = 5×10^6^.

The results in Fig 2 show that the plate width has a very significant effect on the magnitude of the plate vibration frequencies. The results show that the plate vibration frequency decreases with increasing the plate width. This outcome is of significant practical importance and implies that the period of plate vibration increases with increasing plate width. Moreover, the results show that the plate thickness has a significant effect on the magnitude of the plate vibration frequencies. The results show that the vibration frequency increases with increasing plate thickness. This outcome is also of significant practical importance and implies that the period of plate vibration increases with decreasing the plate thickness.

**Fig 3:**
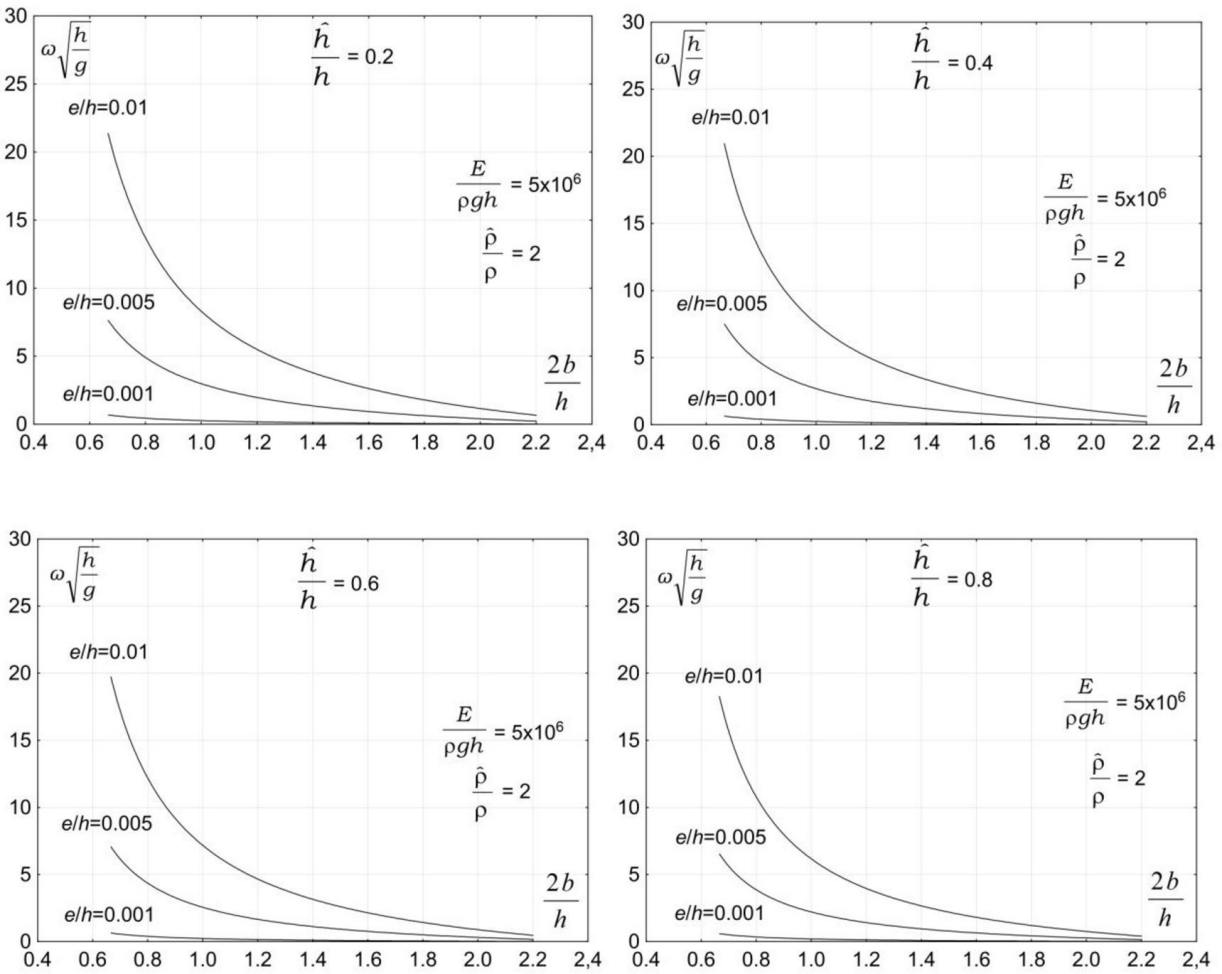
Effect of plate submergence rate on vibration frequency, *E*/*ρ*gh = 5×10^6^$\hat{\rho }/\rho =2$.

The next group of parameters affecting the vibration of a submerged plate is associated with hydrodynamic conditions. Analysis indicates that the main parameter in this group of major practical importance is the rate of the submergence of a plate. Fig 3 demonstrates the dependency of the vibration frequency on the rate of the submergence of a plate. The plots show the dimensionless vibration frequencies, $\omega \sqrt{h/g}$, plotted versus the dimensionless plate length, 2b/h, for three ratios of the plate thickness to the water depth e/h = 0.001, e/h = 0.005, and e/h = 0.01 and four ratios the plate submergence to the water depth $\hat{h}/h=0.2$ , $\hat{h}/h=0.4$, $\hat{h}/h=0.6$, and $\hat{h}/h=0.8$. The results are plotted for moderate values of material density and elasticity modulus, $\hat{\rho }/\rho =2$, and *E*/*ρgh* = 5×10^6^.

The analysis of the plots in Fig 3 shows that the plate vibration frequency decreases with increasing the rate of the submergence of the plate. The plate vibration frequency decreases with increasing the plate submergence rate for all analyzed plate lengths and thicknesses. A detailed analysis indicates that the process of the vibration of a submerged plate is affected by the masses of water vibrating with a plate. A dominant effect on the process of the vibration of a submerged plate has a mass of water above the plate. This explains why the plate vibration frequency decreases with increasing plate submergence rate.

**Fig 4:**
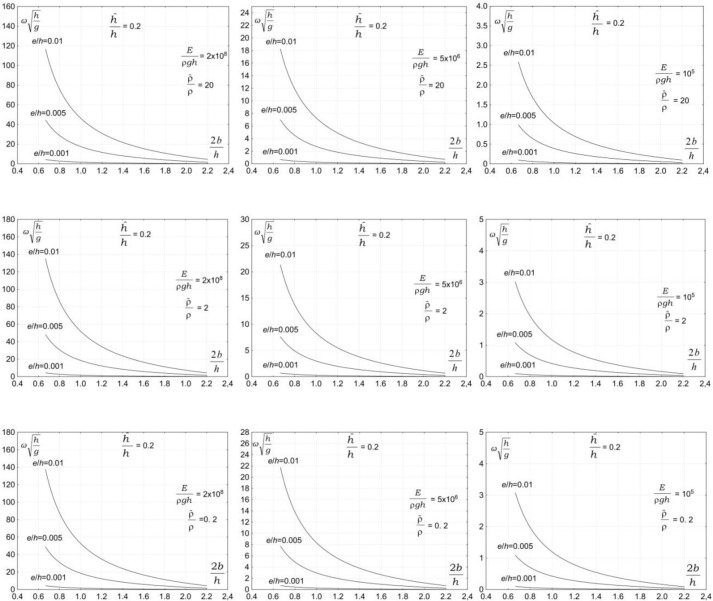
Effects of plate material properties on vibration frequency, $\hat{h}/h=0.2$.

**Fig 5:**
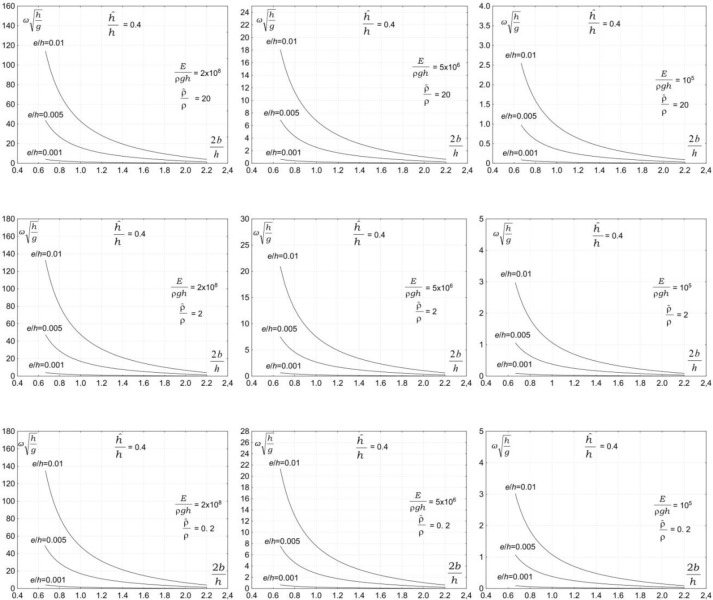
Effects of plate material properties on vibration frequency, $\hat{h}/h=0.4$.

**Fig 6:**
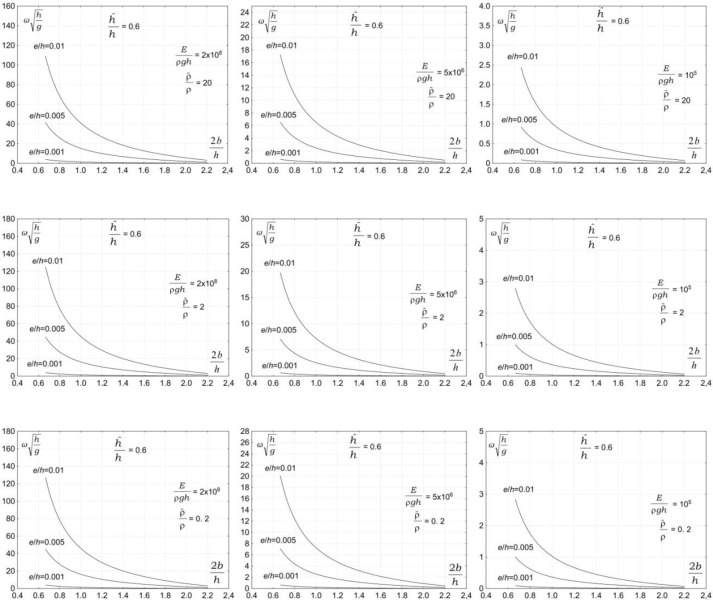
Effects of plate material properties on vibration frequency, $\hat{h}/h=0.6$.

**Fig 7:**
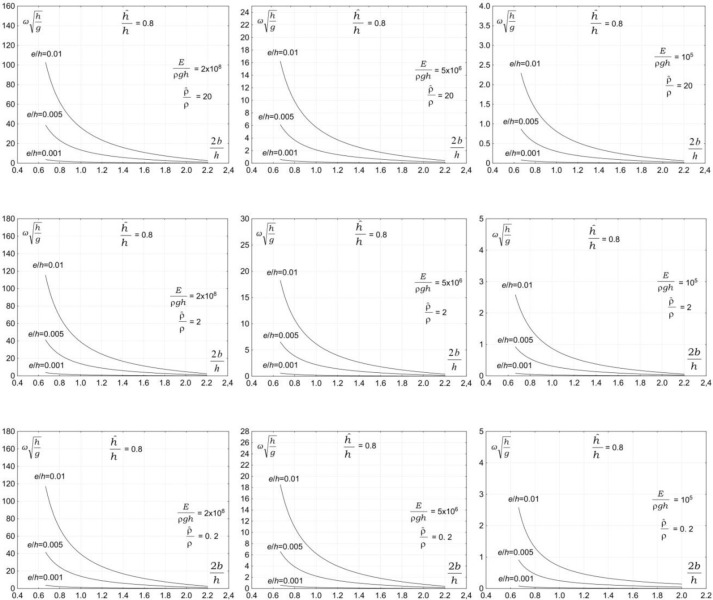
Effects of plate material properties on vibration frequency, $\hat{h}/h=0.8$.

The third group of parameters affecting the vibration of a submerged plate is associated with the properties of plate material. The plate material is expected to have a significant effect on the vibration of a submerged plate. Figs 4–7 show the dependency of the vibration frequency on the properties of plate material. The plots show the dimensionless vibration frequencies, $\omega \sqrt{h/g}$, plotted versus the dimensionless plate length, 2*b/h*, for three ratios of the plate thickness to the water depth *e/h* = 0.001, *e/h* = 0.005, and *e/h* = 0.01, and four ratios the plate submergence to the water depth $\hat{h}/h=0.2$ , $\hat{h}/h=0.4$, $\hat{h}/h=0.6$, and $\hat{h}/h=0.8$. The results are plotted for three values of material density $\hat{\rho }/\rho =0.2$, $\hat{\rho }/\rho =2$, and $\hat{\rho }/\rho =20$ and three values of elasticity modulus, *E*/*ρh* = 10^5,^*E*/*ρh* = 5×10^6^,and *E*/*ρh* = 2×10^8^.

The results in Figs 4–6 show that the properties of plate material have a moderate effect on the vibration frequency. The results show that the plate vibration frequency decreases with increasing the plate material density. A far more significant effect on the vibration frequency has an elasticity modulus. The results show that the plate vibration frequency decreases with decreasing elasticity modulus. For moderate and low values of elasticity modulus, the plate vibration frequency is becoming lower than the frequency of water waves.

This outcome is of significant practical importance because wave frequencies coincide with the natural plate vibration frequencies. The problem is very serious because the vibrations of a plate are amplified by incoming waves, which may lead to resonance. The occurrence of a resonance may lead to the failure of a structure. The analysis shows that the overlapping of plate vibration frequencies and wave frequencies, and in consequence, a resonance, may occur for a very wide range of wave and plate parameters.

## 4. Comparison with experiments

### 4.1. Laboratory experiments

Laboratory experiments were conducted in the hydraulic laboratory at the Institute of Hydro- Engineering, Polish Academy of Sciences, Gdańsk. The wave flume at the Institute of Hydro -Engineering is 64m long, 0.60m wide, and 1.4m deep. The side walls are made of 0.02m thick and 1.4m high glass plates. The wave flume is equipped with a porous wave absorber of length 7.6 m and a slope of 15%. Water waves in the flume are generated by a programmable piston-type wavemaker. The wave flume is equipped with a unique active wave absorption system.

The plate used in laboratory experiments conducted in a wave flume was made from thin stainless steel. The length, width, and thickness of the plate were equal to 0.994m, 0.590m, and 0.004m, respectively. The density of the stainless steel was equal to ρ = 7900 kg/m^3^ and the modulus of elasticity was equal to E = 2.02 10^11^ N/m^2^.

**Fig 8:**
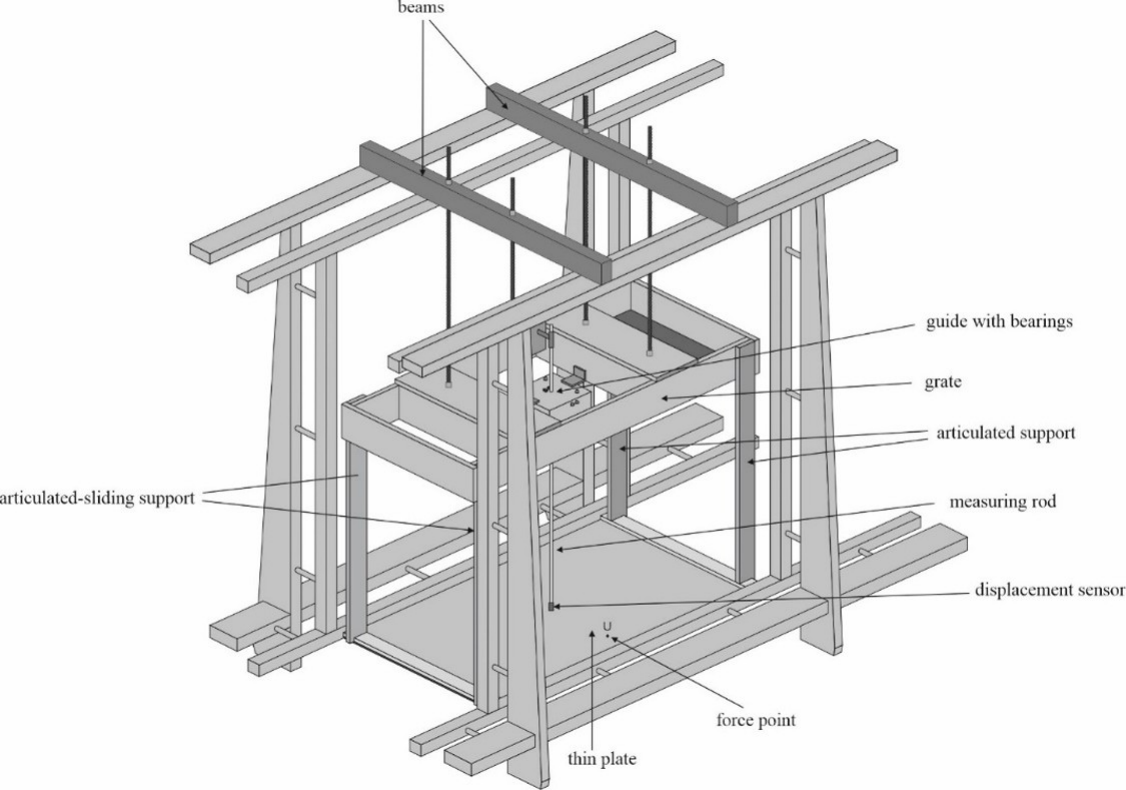
Laboratory model in the channel.

The plate is supported by four front and rear hangers that make the vertical part of the plate supporting system [Fig 8]. The length, width, and thickness of the vertical elements of the plate supporting system were equal to 0.842m, 0.060m, and 0.020m, respectively. The vertical hangers are fixed to the upper plate support system constructed from longitudinal and transverse beams. The plate support system is connected/fastened to the frame of the flume by a system of rods. The system of rods is applied to change the vertical position of the plate.

A vertical displacement gauge/sensor was mounted on the frame supporting the plate. The displacement gauge was installed in the middle of the plate. The gauge vertical movement was secured by a system of bearings. A special edge support mechanism was constructed [by applying bearings] and built into the plate support system to secure the vanishing of deflection and bending moments and to ensure that the constructed physical model represents a simply supported elastic plate.

The model installed in the wave channel is shown in Fig 8. Dimensions and characteristics of the individual elements of the laboratory model are summarized in Table 1.

**Table 1:** Characteristic of the elements of the model.

Model element	Characteristic
thin plate	stainless steel plate dimensions: 590x994x4 mm
flat bars	aluminum, cross-section: 20x60 mm front flat bars: 892 mm rear flat bars: 842 mm
rod	diameter: 6mm
grate	1102x414x100 mm
beams	Dimension: 121x122x372 distance between the beams: 478 mm plywood material
inductive displacement sensor	range: 10 mm, accuracy: 0.01 mm

Laboratory experiments were conducted with the plate installed in a wave flume filled with water, *h* = 0.60m. The experiments were carried out for the plate of different submergence drafts. The first series of experiments were conducted for the draft *d* = 0.20m which was increased in subsequent experiments by 0.01 m up to *d* = 0.58m. For each position of the plate, two tests were carried out with the vibrations initiated by a release of small initial displacements, 0.003 m, and 0.006 m, and then an experiment was conducted with the vibrations initiated by a hit with a rubble-covered bar. Alltogether, 127 experiments were conducted to investigate the effect of a finite water depth on the free vibration of a plate in water.

### 4.2. Theoretical results and experimental data

The analyses of the free vibrations of the plate were conducted by applying a Fourier method [26]. Moreover, the Kalman filters technique was applied to analyze the records of the plate displacements, especially, to decompose the records to determine their dominant components in analogy to a similar technique applied by Sulisz et al. in [27]. Additionally, the free vibrations of the plate were analyzed by employing a recognized technique widely used in the analysis of damped vibrations of a structure [28].

**Fig 9:**
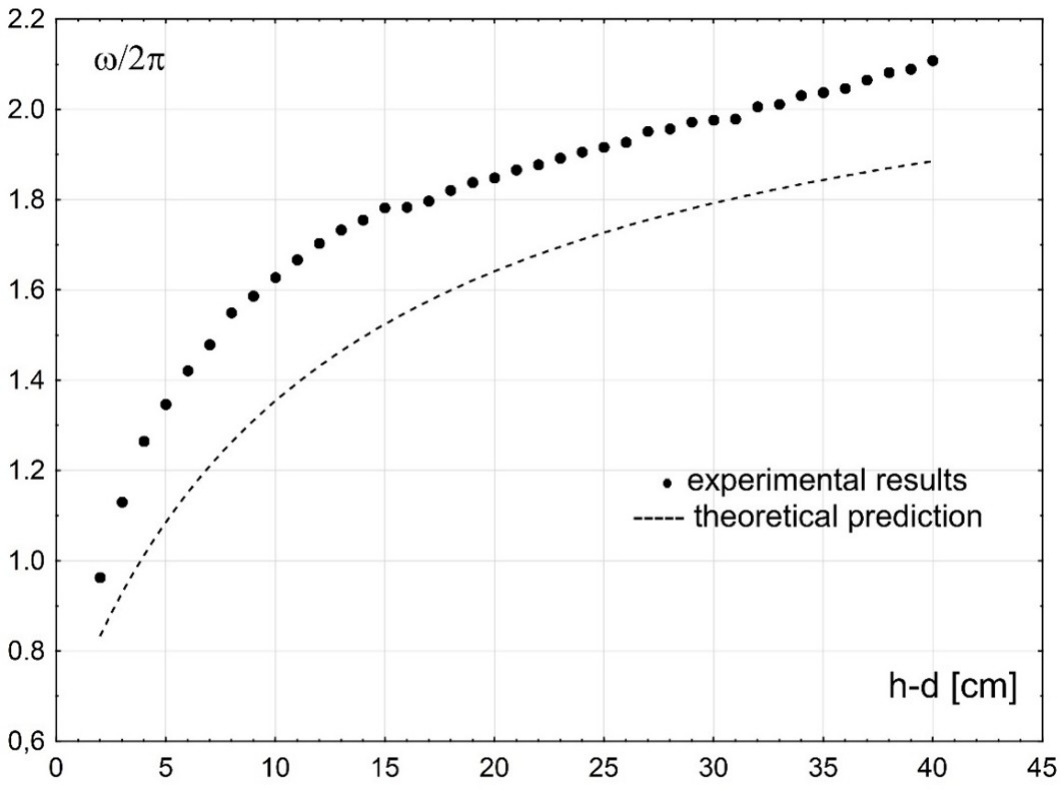
A comparison between theoretical results and experimental data.

A comparison between theoretical results and experimental data is shown in Fig 9. The plots present the theoretical results and the experimental data of the plate vibration frequencies obtained during laboratory experiments. The theoretical results and the experimental data are plotted versus the clearance between the plate and the bed to demonstrate the effect of the submergence rate on the free vibration of a simply-supported plate.

The plots in Fig 9 show that the theoretical results are in reasonable agreement with the experimental data. The derived theoretical model predicts fairly well the free vibration frequencies of a simply-supported submerged horizontal plate. The agreement between theoretical results and experimental data is satisfactory for the whole range of the plate submergence depths considered in laboratory experiments. It is interesting to note that a reasonable agreement is observed between the theoretical results and experimental data even for cases when the clearance between the plate and the bed is becoming small. Some discrepancies between the theoretical results and the experimental data are likely due to simplifications applied in the description of the physical model.

It is worth to noting that laboratory experiments confirmed that the plate vibration frequencies may coincide with the incoming wave frequencies. The overlapping of the plate vibration frequencies with the incoming wave frequency causes resonance. The problem is very serious because the vibrations of a plate are amplified by incoming waves. The occurrence of a resonance amplified by incoming waves may lead to the failure of a structure.

In the conducted analysis, the zero potential boundary condition is applied at the free surface to determine the frequencies of the vibrating plate. For completeness, the wave kinematic boundary conditions were also applied at the free surface to determine the frequencies of the vibrating plate. The results were practically the same as in the present analysis.

## 5. Summary

A theoretical approach was applied to investigate the vibrations of a simply-supported horizontal plate. A boundary-value problem was formulated within potential wave theory and was solved by employing the method of matched eigenfunction expansions. The derived analytical solution was applied to determine flow fields and loads on a plate. The derived forces were applied in a motion equation to predict natural frequencies for a simple-supported horizontal plate vibrating in a fluid.

The derived model was applied to analyze the effect of the geometry of the plate and its physical and material properties on the free vibration of the submerged plate. The effect of flexural plate rigidity and immersion depth has been taken into account.

The results show that the plate width has a very significant effect on the magnitude of the plate vibration frequencies. The results show that the plate vibration frequency decreases with increasing the plate width. Moreover, the results show that the plate thickness has a significant effect on the magnitude of the plate vibration frequencies. The results show that the vibration frequency decreases with decreasing the plate thickness. Moreover, the results show that the vibration frequency depends on the plate draft. The results show that the plate vibration frequency decreases with increasing the plate draft. Analysis indicates that the process of the vibration of a submerged plate is affected by the amount of the masses of water vibrating with a plate. A detailed analysis shows that a dominant effect on the vibration of a submerged plate has a mass of water above the plate, which explains why the plate vibration frequency decreases with increasing plate submergence rate.

The results show that the properties of plate material have a significant effect on the vibration frequencies. The results show that the plate vibration frequency decreases with increasing the plate material density. A far more significant effect on the vibration frequencies has an elasticity modulus. The results show that the plate vibration frequency decreases with decreasing elasticity modulus. For moderate and low values of elasticity modulus, the plate vibration frequency is becoming lower than the frequency of water waves. This outcome is of significant practical importance because wave frequencies overlap with the natural plate vibration frequencies, which may lead to resonance and the failure of a structure. The problem is that the overlap of plate vibration frequencies and wave frequencies occurs for a very wide range of wave and plate parameters.

Laboratory experiments were conducted in a wave flume to verify numerical results. The theoretical results are in reasonable agreement with experimental data.

## Supporting information

S1 Appendix (DOCX)
