# Supplementary material for: Modeling of free vibrations and resonant frequencies of simply-supported submerged horizontal plate
Source: PLoS One. 2024 Mar 1;19(3):e0298290. doi: 10.1371/journal.pone.0298290 (PMC10906898; doi:10.1371/journal.pone.0298290)
Supplement: S1 Appendix — (DOCX) [file pone.0298290.s001.docx]

**Appendix**

Derivation of equations applied to obtain the coefficients of velocity potentials

 (A1)

 (A2)

 (A3)

$\begin{matrix} \int_{-h}^{-d} {}_{1}\varphi_{32x}\left(x=b\right)\cos\alpha_{1m}(z+h)dz=\int_{-h}^{-d} {}_{1}\varphi_{32x}\left(x=b\right)\cos\alpha_{1m}(z+h)dz= \\ =-i\omega\int_{-h}^{-d} \sum_{r=1} E_{1r}\frac{\cosh\beta_{1r}(z +h)}{\sinh\beta_{1r}(h-d)}\text{cos}\beta_{1r}2b\cos\alpha_{1m}(z+h)dz= \\ =i\omega\sum_{r=1} E_{1r}\frac{\text{Cos2b}\beta_{1r}}{\alpha_{1m}^{2}+\beta_{1r}^{2}}\left(\beta_{1r}\cos\alpha_{1m}\left(h-d\right)+\alpha_{1m}\coth\beta_{1r}(h-d)\sin\alpha_{1m}(h-d)\right) \\ \end{matrix}$ (A4) (A5)

 (A6)

$$\begin{matrix} \int_{-h}^{-d} {}_{1}\varphi_{32}\left(x=b\right)\cos\mu_{1m}(z+h)dz=\int_{-h}^{-d} -i\omega\sum_{r=1} \frac{E_{1r}}{\beta_{1r}}\frac{\cosh\beta_{1r}(z+h) }{\sinh\beta_{1r}(h-d)}\sin2\beta_{1r}b\cos\mu_{1m}(z+h)dz= \\ =i\omega\sum_{r=1} E_{1r}\frac{\text{Sin2b}\beta_{1r}}{\beta_{1r}\left(\mu_{1m}^{2}+\beta_{1r}^{2}\right)}\left(\beta_{1r}\cos\mu_{1m}\left(h-d\right)+\mu_{1m}\coth\beta_{1r}(h-d)\sin\mu_{1m}(h-d)\right) \end{matrix}$$

(A7)
